# Natural Product Ginsenoside 25-OCH_3_-PPD Inhibits Breast Cancer Growth and Metastasis through Down-Regulating MDM2

**DOI:** 10.1371/journal.pone.0041586

**Published:** 2012-07-23

**Authors:** Wei Wang, Xu Zhang, Jiang-Jiang Qin, Sukesh Voruganti, Subhasree Ashok Nag, Ming-Hai Wang, Hui Wang, Ruiwen Zhang

**Affiliations:** 1 Department of Pharmaceutical Sciences, School of Pharmacy, Texas Tech University Health Sciences Center, Amarillo, Texas, United States of America; 2 Cancer Biology Center, School of Pharmacy, Texas Tech University Health Sciences Center, Amarillo, Texas, United States of America; 3 Department of Biomedical Sciences, School of Pharmacy, Texas Tech University Health Sciences Center, Amarillo, Texas, United States of America; 4 Institute for Nutritional Sciences, Shanghai Institutes for Biological Sciences, Chinese Academy of Sciences, Shanghai, China; Virginia Commonwealth University, United States of America

## Abstract

Although ginseng and related herbs have a long history of utility for various health benefits, their application in cancer therapy and underlying mechanisms of action are not fully understood. Our recent work has shown that 20(S)-25-methoxyl-dammarane-3*β*, 12*β*, 20-triol (25-OCH_3_-PPD), a newly identified ginsenoside from *Panax notoginseng*, exerts activities against a variety of cancer cells *in vitro* and *in vivo*. This study was designed to investigate its anti-breast cancer activity and the underlying mechanisms of action. We observed that 25-OCH_3_-PPD decreased the survival of breast cancer cells by induction of apoptosis and G1 phase arrest and inhibited the growth of breast cancer xenografts *in vivo*. We further demonstrated that, in a dose- and time-dependent manner, 25-OCH_3_-PPD inhibited MDM2 expression at both transcriptional and post-translational levels in human breast cancer cells with various p53 statuses (wild type and mutant). Moreover, 25-OCH_3_-PPD inhibited *in vitro* cell migration, reduced the expression of epithelial-to-mesenchymal transition (EMT) markers, and prevented *in vivo* metastasis of breast cancer. In summary, 25-OCH_3_-PPD is a potential therapeutic and anti-metastatic agent for human breast cancer through down-regulating MDM2. Further preclinical and clinical development of this agent is warranted.

## Introduction

Although early detection and improvements in surgical operation, chemotherapy, and radiotherapy have led to an increase in patient survival, breast cancer still remains the second leading cause of cancer death in women in North America [1]. There are increasing efforts to develop more effective, less toxic therapeutic agents. The identification of oncogenes involved in the initiation and progression of tumors has facilitated the identification of targets for the development of new anticancer drugs [2]. Several new drugs, small molecules, and monoclonal antibodies directly affecting oncogene products have been developed. Examples of oncogene-based, molecular therapeutics that show promising clinical activity include trastuzumab (Herceptin), imatinib (Gleevec), and gefitinib (Iressa). However, the potential of oncogenes as novel targets for cancer therapy has not been fully realized and many challenges remain, from the validation of novel targets, to the design of specific agents, to the evaluation of these agents in both preclinical and clinical studies.

Natural products provide a rich source for developing novel anticancer agents. Ginseng (*Panax ginseng*) has been used for thousands of years in China and other Asian countries for a variety of diseases. Pre-clinical and clinical studies indicate that ginseng products can be effective and safe anti-cancer agents, as reviewed recently in [3]. The anti-tumor efficacy of ginseng is attributed mainly to the presence of saponins, known as ginsenosides. There is an increasing interest in developing ginseng products as anticancer agents, especially in combination therapy. We have been interested in evaluating the anticancer activity of ginseng and related herb medicines and have recently isolated 20(S)-25-methoxyl-dammarane-3*β*, 12*β*, 20-triol (25-OCH_3_-PPD), a natural product from *P. notoginseng*, and demonstrated its cytotoxicity against a variety of cancer cells [4]–[9]. Thus far, 25-OCH_3_-PPD is one of the most active ginsenosides that have been evaluated for cancer-specific effects [5], [9]. For example, we carried out a side-by-side comparison for *in vitro* anticancer activity in various cancer cell lines (breast, prostate, pancreatic, lung cancers and glioma) among three ginsenosides, 25-OCH_3_-PPD, PPD, and Rg_3_. Based on IC_50_ values, 25-OCH_3_-PPD showed 5–10-fold better activities than PPD and 10–100-fold better activities than Rg_3,_ a marketed drug in China [4]. *In vivo* anticancer activities of 25-OCH_3_-PPD have been demonstrated in mouse xenograft models of human cancers of lung [7], pancreas [8], and prostate [9]. Several potential molecular targets for this compound have been suggested, including genes regulating cell cycle and apoptosis [7]–[9].

**Figure 1:**
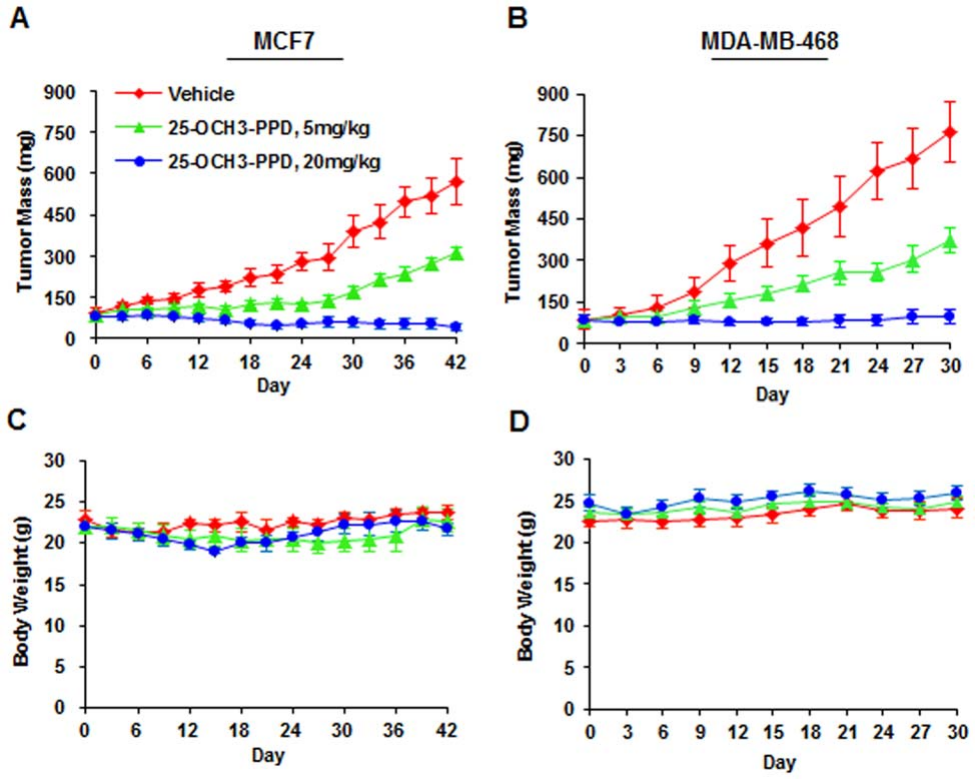
25-OCH_3_-PPD inhibits the growth of breast cancer xenograft tumors. 25-OCH_3_-PPD was administered by i.p. injection at doses of 5 or 20 mg/kg/d, 5 days/wk for 6 (MCF7 (A)) or 4 weeks (MDA-MB-468 (B)). The growth of tumors was monitored. The body weights of animals were also monitored as a surrogate marker for toxicity in MCF7 (C) and MDA-MB-468 (D) xenograft models.

**Figure 2:**
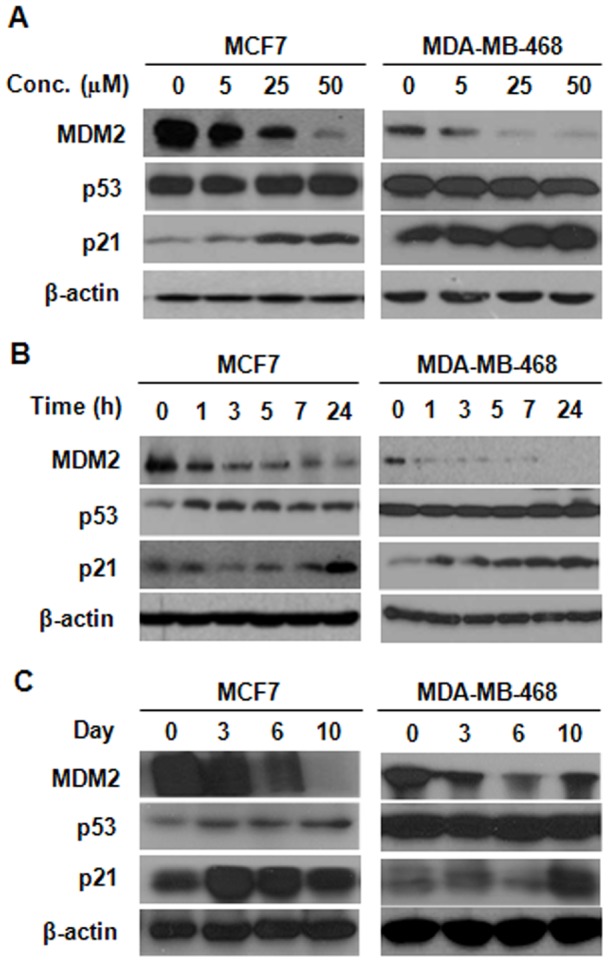
25-OCH_3_-PPD decreases MDM2 expression in a dose- and time-dependent manner in human breast cancer cells. MCF7 (p53 wild type) and MDA-MB-468 (p53 mutant) cells were treated with various concentrations of 25-OCH_3_-PPD for 24 h (A) or with 25 µM 25-OCH_3_-PPD for various periods (B). (C) 25-OCH_3_-PPD also inhibits MDM2 expression *in vivo*. The tumor-bearing animals were treated with 25-OCH_3_-PPD (20 mg/kg/d, 5 days/wk) and tumors were removed at indicated times. The protein levels of MDM2, p53, and p21 in tumor tissue homogenates were analyzed by Western blotting.

**Figure 3:**
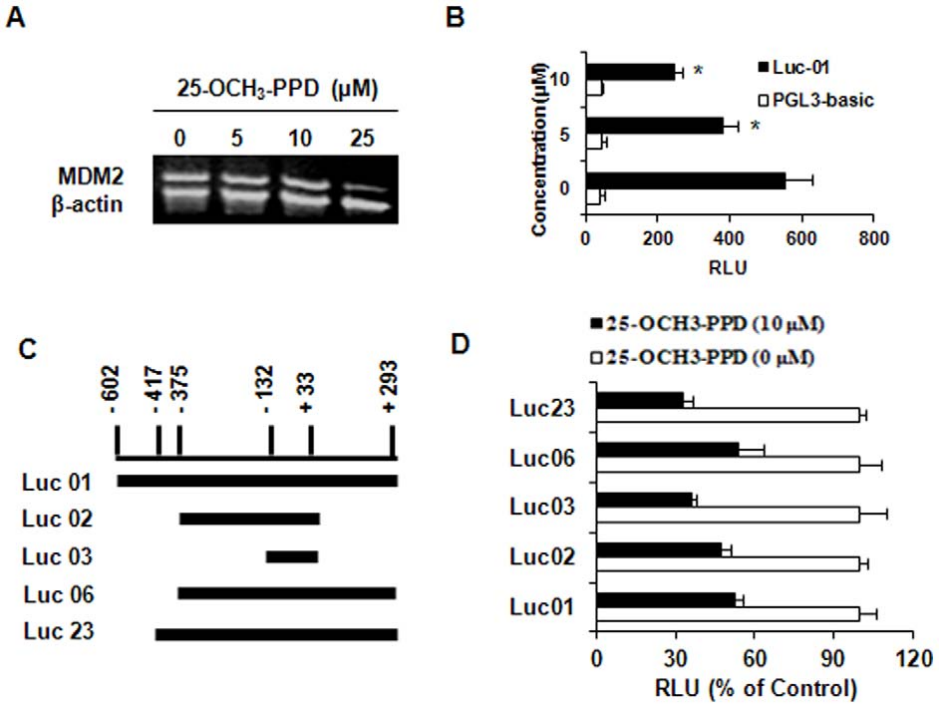
25-OCH_3_-PPD inhibits MDM2 transcription. (A) MCF7 cells were treated with various concentrations of 25-OCH_3_-PPD for 24 h. MDM2 mRNA was co-amplified with β-actin mRNA. The relative levels of MDM2 were normalized to that of β-actin. (B) MCF7 cells were co-transfected with full-length MDM2 P2 promoter luciferase vectors and a Renilla luciferase reporter, followed by incubation with 25-OCH_3_-PPD for 24 h. The MDM2 luciferase activity was detected using the Dual-Luciferase Reporter Assay System. All the analyses were performed in triplicate. (C) Structures of full-length and deleted MDM2 P2 promoter. (D) The effects of 25-OCH_3_-PPD (10 µM) on the activity of various MDM2 luciferase reporters were analyzed using the same procedure as above (B). Luciferase activities were plotted as percentages of the control. * P<0.01.

**Figure 4:**
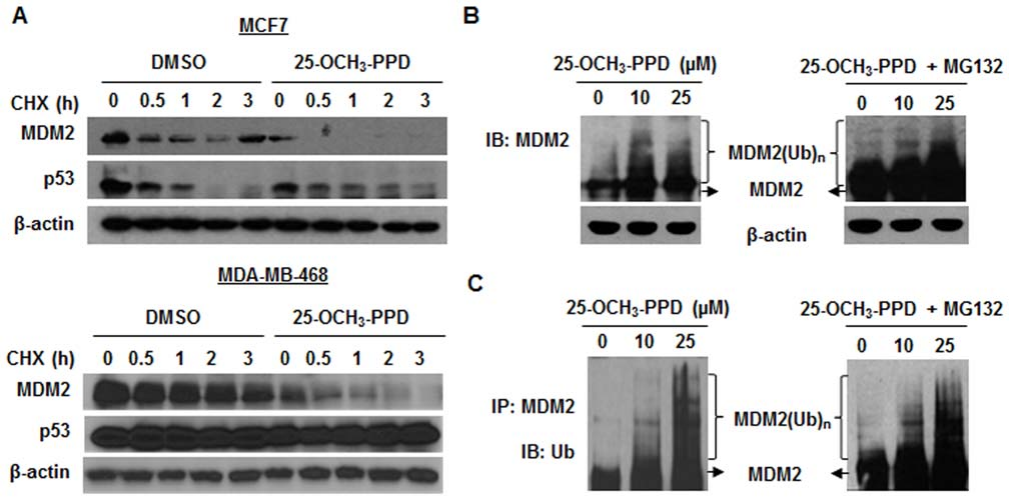
25-OCH_3_-PPD destabilizes MDM2 protein. (A) MCF7 and MDA-MB-468 cells were treated with DMSO or 25-OCH_3_-PPD (25 µM) followed by exposure to protein synthesis inhibitor cycloheximide (CHX, 10 µg/mL). MDM2 levels were detected by Western blotting at indicated times after exposure to CHX. (B) MCF7 cells were transfected with MDM2 and ubiquitin plasmids followed by treatment with various concentrations of 25-OCH_3_-PPD for 24 h. Cells were harvested (left panel) or exposed to proteasome inhibitor MG132 (25 μM) for additional 6 h (right panel). Ubiquitinated MDM2 was detected by immunoblotting. (C) The cell lysates of MCF7 cells treated as above were immunoprecipitated with anti-MDM2 antibody. The ubiquitinated MDM2 was detected using anti-ubiquitin antibody.

**Figure 5:**
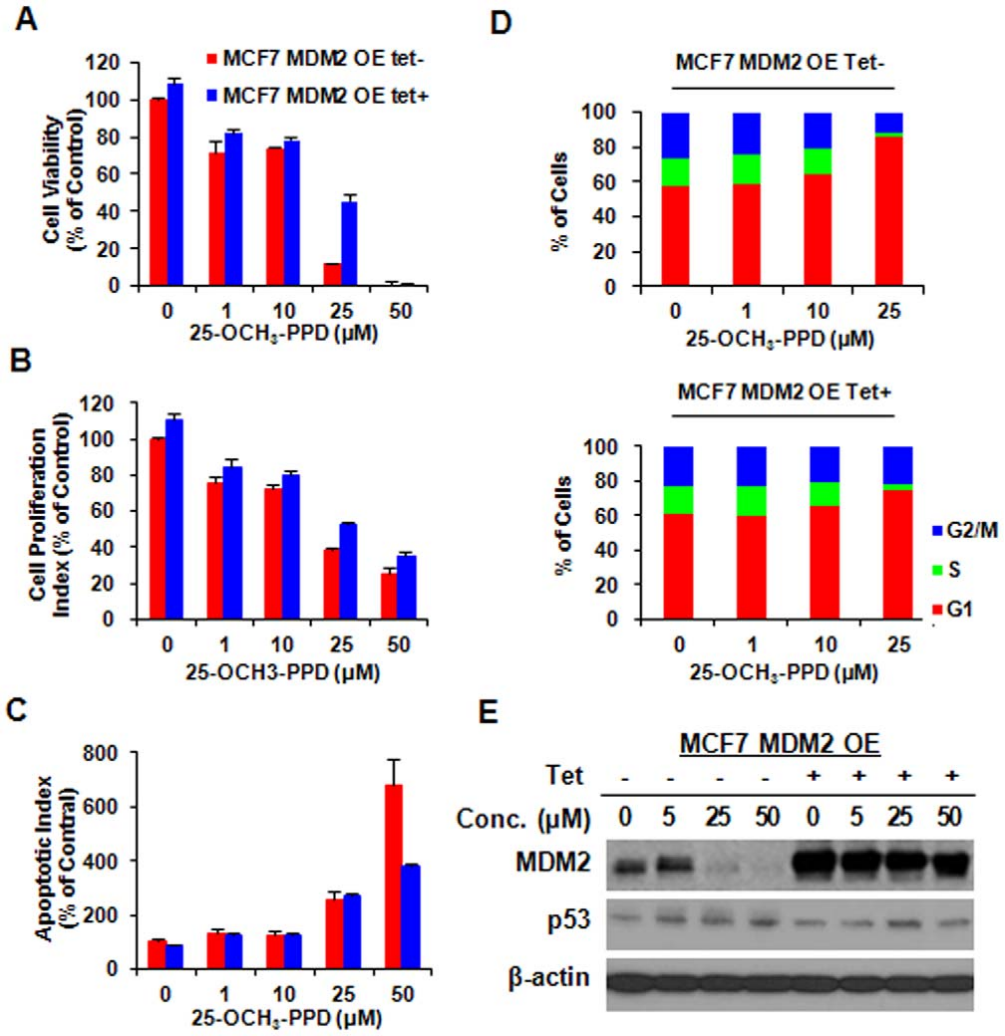
MDM2 overexpression reduces cell response to 25-OCH_3_-PPD treatment. MCF7 MDM2-inducible cells were incubated with (Tet+) or without tetracycline (Tet-) for 12 h, followed by exposure to various concentrations of 25-OCH_3_-PPD for various times: (A) 72 h for analysis of cell viability using MTT assay; (B) 24 h for analysis of cell proliferation using BrdUrd assay; (C) 48 h for apoptosis analysis using Flow Cytometry; and (D) 24 h for cell cycle analysis. (E) 25-OCH_3_-PPD inhibits MDM2 expression in MCF7 inducible cells without tetracycline. MDM2 overexpression was confirmed in MCF7 inducible cell line treated with tetracycline, which reversed the effect of 25-OCH_3_-PPD.

**Figure 6:**
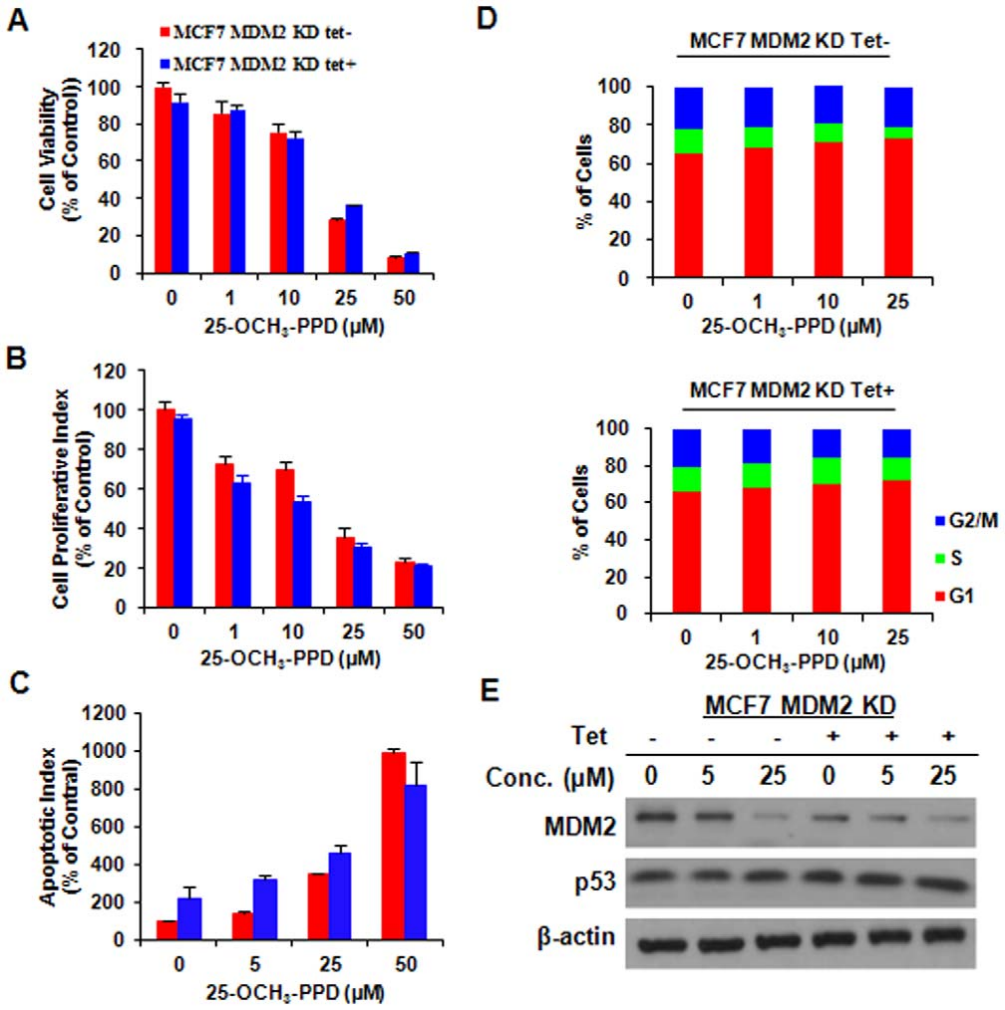
MDM2 knockdown reduces cell response to 25-OCH_3_-PPD treatment. MCF7 MDM2-inducible cells were incubated with (Tet+) or without tetracycline (Tet-) for 12 h, followed by exposure to various concentrations of 25-OCH_3_-PPD for various times: (A) 72 h for analysis of cell viability using MTT assay; (B) 24 h for analysis of cell proliferation using BrdUrd assay; (C) 48 h for apoptosis analysis using Flow Cytometry; and (D) 24 h for cell cycle analysis. (E) 25-OCH_3_-PPD inhibits MDM2 expression in MCF7 inducible cells with or without tetracycline. MDM2 knockdown was confirmed in MCF7 inducible cell line treated with tetracycline, which reversed the effect of 25-OCH_3_-PPD.

**Figure 7:**
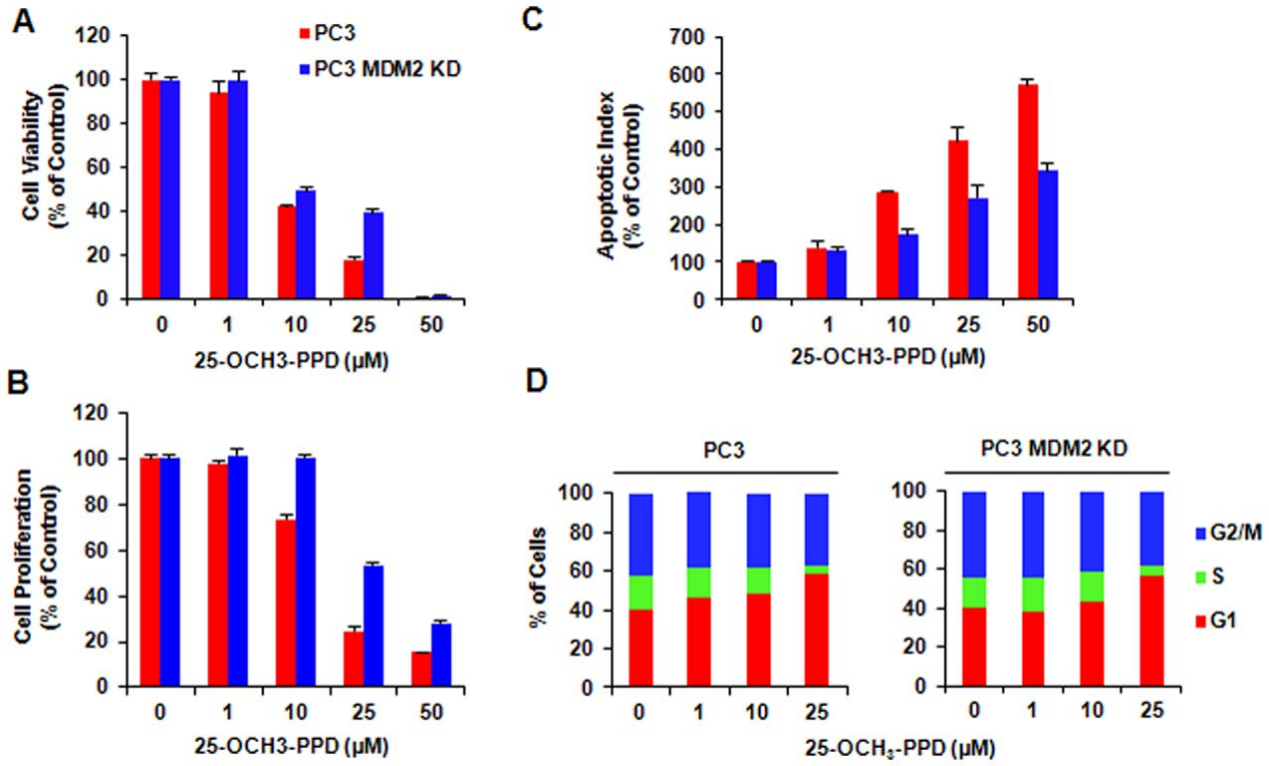
MDM2 knockdown reduces cell sensitivity to 25-OCH_3_-PPD treatment. PC3 cells and PC3 MDM2 KD cells were treated with various concentrations of 25-OCH_3_-PPD for various times: (A) 72 h for analysis of cell viability using MTT assay; (B) 24 h for analysis of cell proliferation using BrdUrd assay; (C) 48 h for apoptosis analysis using Flow Cytometry; and (D) 24 h for cell cycle analysis.

**Figure 8:**
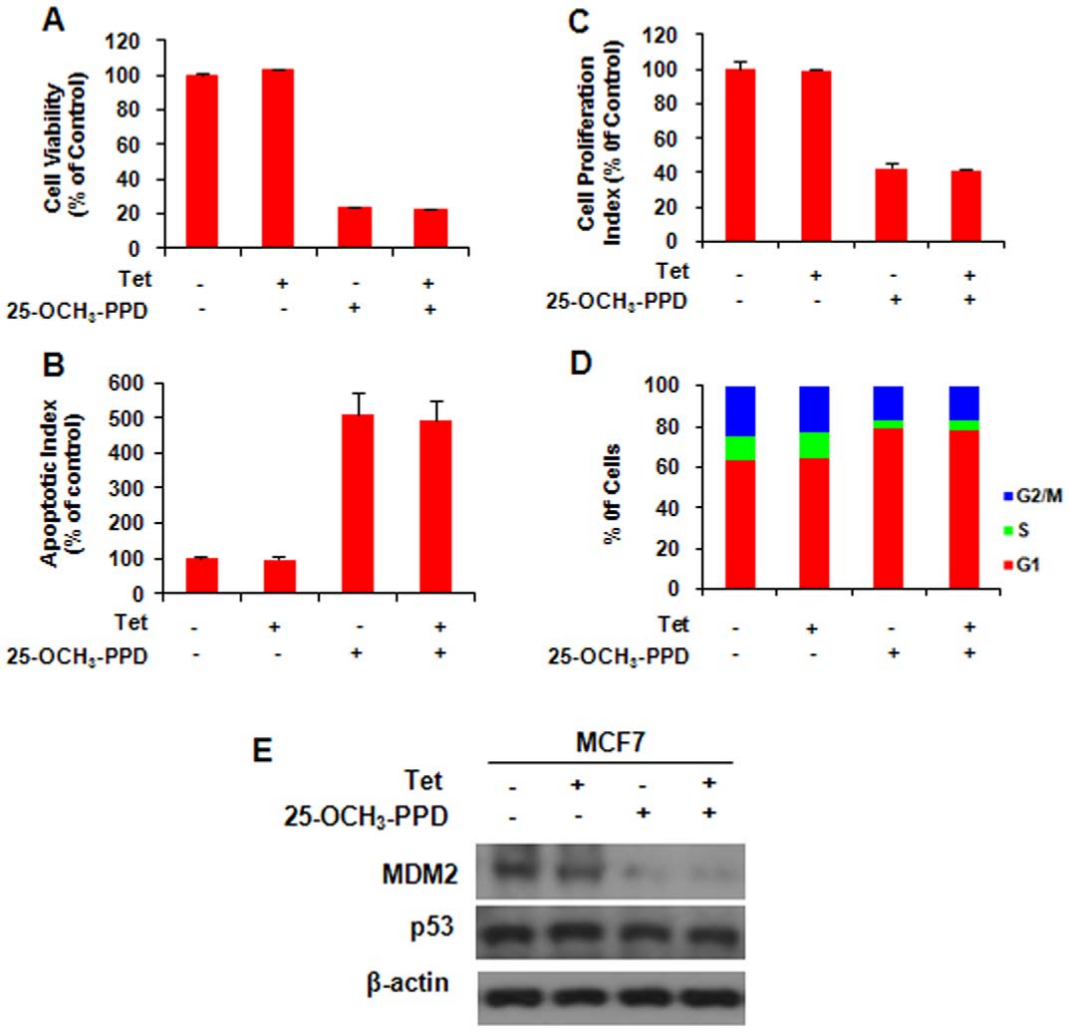
Tetracycline treatment does not affect parent MCF7 cells. MCF7 cells were incubated with (Tet+) or without tetracycline (Tet-) for 12 h, followed by exposure to 25-OCH_3_-PPD (25 µM) for various times: (A) 72 h for analysis of cell viability using MTT assay; (B) 24 h for analysis of cell proliferation using BrdUrd assay; (C) 48 h for apoptosis analysis using Flow Cytometry; and (D) 24 h for cell cycle analysis. (E) 25-OCH_3_-PPD inhibits MDM2 expression in MCF7 cells with or without tetracycline.

**Figure 9:**
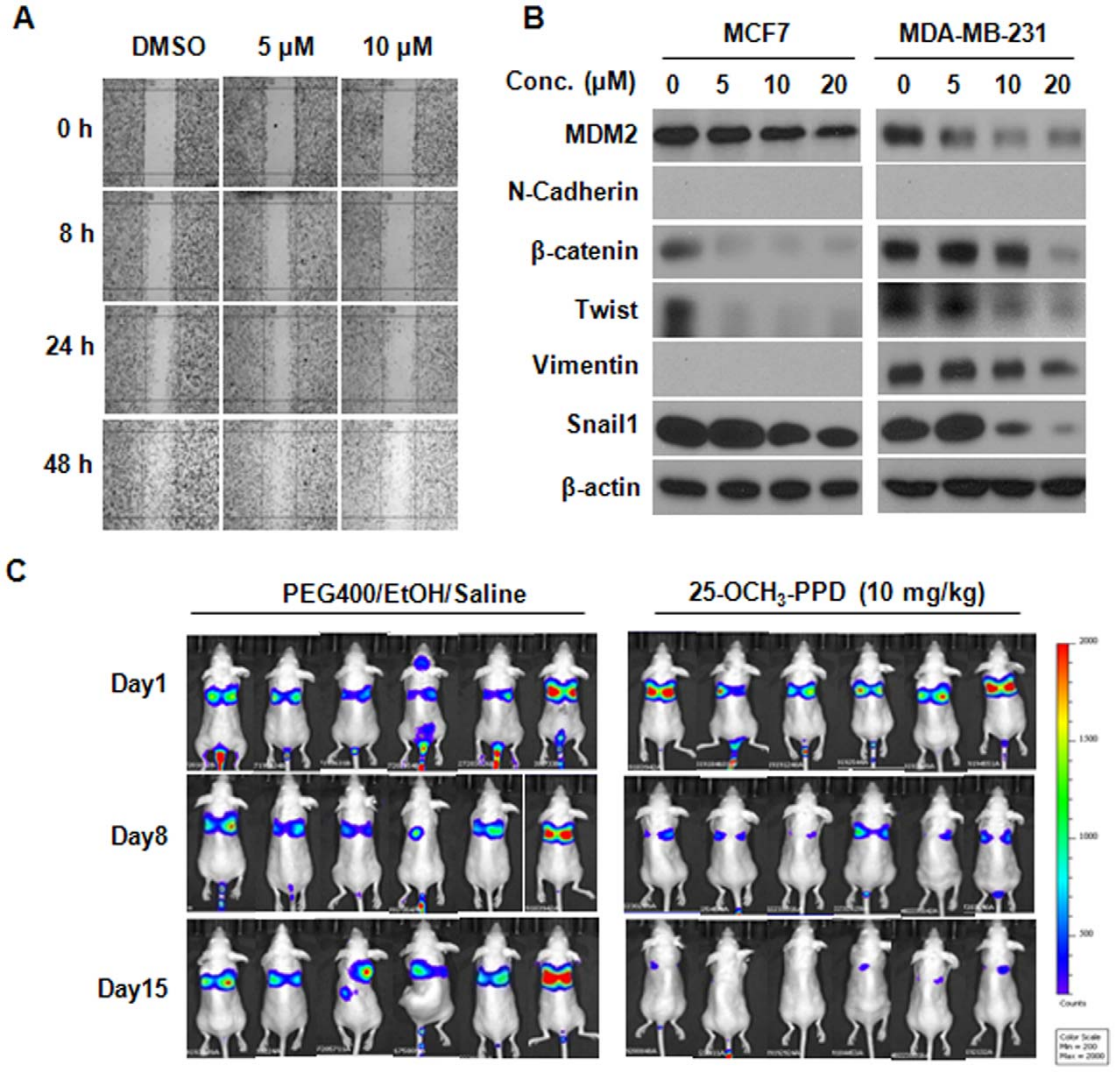
25-OCH_3_-PPD inhibits cell migration *in vitro* and metastasis *in vivo*. (A) Wound healing assay in MDA-MB-231 cells. After cell growth reached confluence, a scratch was made, and then the cells were incubated with various concentrations of 25-OCH_3_-PPD for indicated times. The closure of the scratch was imaged. (B) Effects of 25-OCH_3_-PPD on the expression of EMT markers in human breast cancer cells. Cells were treated with various concentrations of 25-OCH_3_-PPD for 48 h. The expression of EMT markers was analyzed by Western blotting. (C) *In vivo* metastasis assay. 1×10^6^ of MDA-MB-231-Luc cells were intravenously injected into a tail vein of nude mice. 25-OCH_3_-PPD was administered by i.p. injection at a dose of 10 mg/kg/d, 5 days/wk for 2 weeks. The luciferase signals were determined and photographed using IVIS *in vivo* image system on Days 1, 8, and 15.

Oncogenes have been shown to be potential molecular targets for cancer treatment and prevention. As one of the most studied oncogenes, the MDM2 oncogene plays an important role in cancer development, progression, and treatment [10]. The MDM2 oncogene is amplified and overexpressed in various human cancers. It has been suggested that high MDM2 levels are associated with poor prognosis of several human cancers [11]. The MDM2 oncoprotein is a negative regulator of p53 and there is an auto-regulatory loop between MDM2 and p53 [12]–[14]. We and others have provided evidence supporting that MDM2 could be a target for cancer therapy [15], [16]. Various MDM2 inhibitors, including antisense oligonucleotides, synthetic small molecules, and natural products, have shown anti-cancer activity *in vitro* and *in vivo* [17]–[21]. In our previous studies, we have shown that 25-OCH_3_-PPD inhibits MDM2 in cancer cells [7]–[9], although the detailed mechanisms are not fully understood.

Considering that 25-OCH_3_-PPD has potent *in vitro* activity in breast cancer cells [4] and *in vitro* and *in vivo* efficacy in other cancers [7]–[9], little *in vitro* cytotoxicity to normal cells [9], and little or no *in vivo* toxicity at the tested doses [7]–[9], we speculated that it can be an effective and safe agent for breast cancer treatment, with prolonged administration. We were particularly interested in addressing the possibility of using this compound for prevention and treatment of metastatic breast cancer. In the present study, after demonstration of its anti-breast cancer activity *in vivo*, we attempted to explore the molecular mechanisms for the effects of 25-OCH_3_-PPD on both primary and metastatic breast cancers, showing MDM2 to be a major target. MDM2 has been linked to cancer development, progression, and metastasis. Therefore, we believe that our results should provide a basis for future development of this ginsenoside as a novel anti-breast cancer agent for both primary and metastatic diseases.

## Results

### 25-OCH_3_-PPD inhibits the growth of human breast cancer *in vivo*

Since we have previously shown the *in vitro* cancer-specific activity of 25-OCH_3_-PPD [4], [7]–[9], we decided to first determine the therapeutic effectiveness of this compound against breast cancer *in vivo*. The nude mice bearing MCF7 or MDA-MB-468 xenograft tumors were treated with 25-OCH_3_-PPD by i.p. injection at doses of 5 or 20 mg/kg/d for 6 weeks or 4 weeks, respectively. As shown in Fig. 1A, two dose levels of 25-OCH_3_-PPD (5 and 20 mg/kg) inhibited MCF7 xenograft tumor growth by about 60% and 90%, respectively (P<0.01). The same figures in MDA-MB-468 xenograft tumor model were 50% and 87%, respectively (P<0.01) (Fig. 1B). There were no significant differences in body weights between control and 25-OCH_3_-PPD treated animals (Figs. 1C and 1D). Taken together, these data demonstrate that 25-OCH_3_-PPD has significant anti-breast cancer activity *in vivo*.

### 25-OCH_3_-PPD inhibits MDM2 expression

The effects of 25-OCH_3_-PPD on MDM2 expression were analyzed in MCF7 (wild type p53) and MDA-MB-468 (mutant p53) cells. The Western blot results showed that MDM2 protein levels were decreased by 25-OCH_3_-PPD treatment, in a dose- and time-dependent manner (Figs. 2A and 2B). In MCF7 cells, most likely as a result of the inhibition of MDM2, the wide-type p53 protein level was elevated. In both MCF7 and MDA-MB-468 cells, p21^Waf1/CIP1^ protein levels were also elevated as a result of MDM2 inhibition (Fig. 2A and 2B). The similar results were observed *in vivo*. The MDM2 protein levels were decreased in both models, alongside with increased p21^ Waf1/CIP1^ levels (Fig. 2C). The p53 levels in treated MCF7 tumors were elevated but mutant p53 levels in MDA-MB-468 tumors unchanged (Fig. 2C). In brief, the *in vitro* and *in vivo* data suggest that MDM2 is a target for 25-OCH_3_-PPD, resulting in the modulation of p53 and p21 ^Waf1/CIP1^ expression.

### 25-OCH_3_-PPD suppresses MDM2 transcription

To explore the possible mechanisms for 25-OCH_3_-PPD-induced MDM2 inhibition, we treated MCF7 cells with various concentrations of 25-OCH_3_-PPD for 24 h and determined the MDM2 mRNA levels by semi-quantitative RT-PCR. As shown in Fig. A, the MDM2 mRNA levels were decreased by 25-OCH_3_-PPD in a dose-dependent manner. To further determine how 25-OCH_3_-PPD reduces MDM2 transcription, we transfected MCF7 cells with the full-length MDM2 P2 promoter luciferase reporter (MDM2-Luc01) or control vector. As shown in Fig. 3B, the luciferase activity of MDM2 promoter was decreased by 25-OCH_3_-PPD in a dose-dependent manner (P<0.01). To further identify the response elements on MDM2 P2 promoter, a series of deletions of the MDM2 luciferase reporter were used (Fig. 3C). As shown in Fig. 3D, compared with control groups for each reporter, all the deletions (Luc 02, 03, 06 and 23) showed similar response to that of full-length reporter (Luc 01). There are some important transcription factor binding sites within these regions (such as ETS, AP1, MEF2 and NFAT). Future study needs to fine tune the exact binding sites for 25-OCH_3_-PPD.

### 25-OCH_3_-PPD destabilizes MDM2 protein

The effects of 25-OCH_3_-PPD on MDM2 expression were also evaluated at the post-translational level. Cells were exposed to 25 µM of 25-OCH_3_-PPD or vehicle control for 24 h, followed by addition of the protein synthesis inhibitor, cycloheximide (CHX, 10 μg/mL) and analyzed at different times. As shown in Fig. 4A, 25-OCH_3_-PPD promoted the turnover of MDM2 protein in both MCF7 and MDA-MB-468 cells. To elucidate the possible underlying mechanisms for decreased MDM2 protein stability by 25-OCH_3_-PPD, MCF7 cells were co-transfected with MDM2 and ubiquitin plasmids followed by treatment with 25-OCH_3_-PPD for 24 h. 25-OCH_3_-PPD treatment led to an increase in the ubiquitination of MDM2 as measured by Western blotting (Fig. B, left panel). Co-treatment with proteasome inhibitor MG132 (25 μM) for additional 6 h also showed that 25-OCH_3_-PPD promoted MDM2 ubiquitination (Fig. B, right panel). The effect of 25-OCH_3_-PPD on MDM2 ubiquitination was further confirmed by immunoprecipitation assay in a separate experiment (Fig. C).

### MDM2 overexpression and knockdown reduce the antitumor activities of 25-OCH_3_-PPD *in vitro*

We previously observed that 25-OCH_3_-PPD decreased cell survival, inhibited cell proliferation, induced cell apoptosis, and led to G1 cell cycle arrest in breast cancer cells [5]. In this study, we first overexpressed MDM2 using Tet-on inducible system in MCF7 cells and further determined the target effect of 25-OCH_3_-PPD on MDM2. As shown in Fig. 5, compared with the parent cells, MDM2 overexpression reversed the cellular response to 25-OCH_3_-PPD, including cell survival (Fig. 5A), apoptosis (Fig. 5C), and G1 cell cycle arrest (Fig. 5D), but not cell proliferation (Fig. 5B), The effects of 25-OCH_3_-PPD on the MDM2 protein level were confirmed in MCF7 inducible cell line without tetracycline treatment (Tet-). Overexpression of MDM2 in the tetracycline-treated inducible cells (Tet+) significantly reduced the effects of 25-OCH_3_-PPD on MDM2 (Fig. 5E), demonstrating that MDM2 is a target for 25-OCH_3_-PPD. To further validate the fact that 25-OCH_3_-PPD targets MDM2 to exert its anti-cancer activities, we compared the effects of 25-OCH_3_-PPD on parent and MDM2 knockdown (KD) MCF7 inducible cells. As shown in Figure 6, similar results were observed. We further confirmed this effect in another pair of cancer cell lines (PC3 and PC3 MDM2 KD) with different p53 status (p53 null) (Fig. 7), removal of MDM2 also inhibited the effects of 25-OCH_3_-PPD, including cell survival (Fig. 7A), cell proliferation (Fig. 7B), apoptosis (Fig. 7C), and G1 cell cycle arrest (Fig. 7D). We also demonstrated that tetracycline treatment had no effect on MCF7 cells and did not overcome the anti-cancer effects of 25-OCH_3_-PPD (Fig. 8).

### 25-OCH_3_-PPD inhibits cell migration *in vitro* and metastasis *in vivo*

Considering that MDM2 has been indicated to correlate with cancer metastasis [22], we next examined whether the MDM2 inhibition by 25-OCH_3_-PPD has a role in breast cancer metastasis. We first performed the wound healing assay to assess the effects of 25-OCH_3_-PPD on cell migration *in vitro*. MDA-MB-231 cells were treated with 5 or 10 µM 25-OCH_3_-PPD for various times. As shown in Fig. 9A, control cells displayed significantly rapid wound closure, tending to protrude into the wound site. 25-OCH_3_-PPD treatment inhibited cell migration, especially at 10 µM. We next analyzed the effects of 25-OCH_3_-PPD on the epithelial-to-mesenchymal transition (EMT) markers. In a dose-dependent manner, 25-OCH_3_-PPD treatment markedly decreased the levels of β-catenin, Twist, Vimentin, and Snail1 proteins in MDA-MB-231 cells (Fig. 9B). 25-OCH_3_-PPD also dose-dependently inhibited the expression of β-catenin, Twist, and Snail1 proteins in MCF7 cells (Fig. 9B). However, MCF7 cells exhibited no N-cadherin and Vimentin expression (Fig. 9B). To further demonstrate the *in vivo* anti-metastatic ability of 25-OCH_3_-PPD, we utilized the MDA-MB-231-Luc metastatic model to monitor distant metastases to the lungs using *in vivo* imaging system. As shown in Fig. 9C, 25-OCH_3_-PPD significantly inhibited lung metastases of MDA-MB-231-Luc cells compared to vehicle control on Days 8 and 15. These results suggest that 25-OCH_3_-PPD inhibits cell metastasis both *in vitro* and *in vivo*.

## Discussion

In this study, we have demonstrated several important points: 1) 25-OCH_3_-PPD suppresses the growth of breast cancer *in vivo*; 2) 25-OCH_3_-PPD inhibits MDM2 expression at both transcriptional and post-translational levels; 3) the inhibition of MDM2 by 25-OCH_3_-PPD is essential for its anti-tumor activities; and 4) 25-OCH_3_-PPD inhibits breast cancer cell migration *in vitro* and metastasis *in vivo*. Our previous studies have demonstrated that 25-OCH_3_-PPD decreases cell viability, inhibits proliferation, induces apoptosis, and arrests cells in the G1 phase in other type of cancer cells [7]–[9]. 25-OCH_3_-PPD modulates the levels of several proteins related to apoptosis and cell proliferation, including cleaved caspases-3, −8, and −9 and, cleaved PARP, Bax, p21, and p27 [7]–[9]. The treatment of 25-OCH_3_-PPD results in a decrease in Bcl-2, cyclin D1, cdks 2, 4 and 6, E2F1 and MDM2 [7]–[9].

Considering that MDM2 is linked to several of these findings [10]–[16], we decided to focus on the mechanism of MDM2 inhibition in this study. MDM2 plays an essential role in cancer development and progression [12]. MDM2 overexpression has been correlated with poor prognosis, increased metastasis and increased aggressiveness of human cancers [10]. MDM2 is suggested to be a potential target for human cancer therapy [15]. The expression of MDM2 is induced by p53 [23] and MDM2 binds to p53 with high affinity and inhibits its transcriptional activity [24], indicating that MDM2 functions as a negative feedback regulator of p53. Thus, MDM2 and p53 form an elegant auto-regulatory feedback loop in which the two proteins mutually control each other's cellular level. In addition, MDM2 overexpression abrogates the ability of p53 to induce cell cycle arrest and apoptosis [25], [26]. MDM2 also enhances the degradation of p53 [27], [28] suggesting that it can regulate p53 functions through multiple mechanisms. Many published studies suggest that overexpression of MDM2 is associated with inactivation of wild-type p53 [29]–[31]. In addition, the MDM2 oncoprotein has also been shown to have p53-independent activity. For example, MDM2 binds to and interacts with pRb [31], E2F1 [32], and p21 [33]. This is important because many malignant tumors show mutant p53 status. In fact, in a transgenic mouse model, overexpression of MDM2 predisposes the mice to spontaneous tumor formation both in the presence and absence of functional p53, indicating a p53-independent role of MDM2 in tumorigenesis [34], [35]. MDM2 is stabilized by mutant p53 [35] and the half-life of MDM2 is prolonged in some p53 mutant leukemia cell lines [36].

In the present study, we found that 25-OCH_3_-PPD down-regulated MDM2 expression at both transcriptional and post-translational levels, regardless of p53 status. At the transcriptional level, several positive responsive elements on the MDM2 P2 promoter were indicated. There are some important transcription factor binding sites within these regions; for example, ETS1 and AP1 regulate MDM2 transcription, independent of p53 [37]. Future experiments need to define the exact responsive elements for 25-OCH_3_-PPD. At the post-translational level, 25-OCH_3_-PPD destabilized MDM2 protein by promoting its ubiquitination. Further investigation is needed to examine how 25-OCH_3_-PPD affects MDM2 ubiquitination and subsequent degradation pathway(s). MDM2 performs its oncogenic roles through p53-dependent and -independent mechanisms. MDM2 may directly interact with p53 targets (such as p21) to promote cancer development and progression. Down-regulation of MDM2 by 25-OCH_3_-PPD in p53 wildtype cells may enhance the stability, transactivity and functions of p53. In p53 mutant cells, MDM2 inhibition by 25-OCH_3_-PPD may trigger the activation of downstream molecules independent of p53.

To further validate that MDM2 is a major target of 25-OCH_3_-PPD, inducible MDM2 overexpression and knockdown MCF7 cells, PC3 and PC3 MDM2 KD (p53 *null*) cells were used in this study. We demonstrated that the MDM2-overexpressing and MDM2-knockdown cells were less sensitive to the compound, showing resistance to apoptosis and cell cycle arrest, thus supporting the importance of MDM2 inhibition in the anti-breast cancer activities of 25-OCH_3_-PPD.

EMT is believed to be associated with drug resistance and cancer metastasis [38], [39]. Previous studies have shown that MDM2 promotes cell motility and invasiveness [40] and MDM2 overexpression correlates with late stage metastatic breast cancer [22]. MDM2 inhibition reduces the expression of EMT markers and decreases the migration of cancer cells [22], [41]. In this study, we demonstrated that 25-OCH_3_-PPD treatment suppressed cell metastasis *in vitro* and *in vivo* and altered the expression of EMT-related proteins. Therefore, we propose that 25-OCH_3_-PPD inhibits cancer cell motility through inhibiting MDM2.

In summary, our preclinical data indicate that 25-OCH_3_-PPD, a novel MDM2 inhibitor, is a potential therapeutic and anti-metastatic agent for human breast cancer. Evidences supporting the notion include the efficacy of 25-OCH_3_-PPD in breast cancer models (regardless of p53 status), favorable safety profiles at effective doses, and significant anti-metastasis effects. Further pre-clinical and clinical studies are warranted to provide a rationale for the development of 25-OCH_3_-PPD as a novel chemotherapy for breast cancer.

## Materials and Methods

### Chemicals, reagents, and plasmids

The identity and purity of 25-OCH_3_-PPD was established previously [5], [9]. All chemicals and solvents used were of the highest analytical grade available. Cell culture supplies and media, fetal bovine serum (FBS), phosphate-buffered saline (PBS), sodium pyruvate, non-essential amino acids (NEAA), and penicillin-streptomycin were obtained from Invitrogen (Carlsbad, CA). Anti-human MDM2 (SMP14) and p21 (C19) antibodies were purchased from Santa Cruz Biotechnology (Santa Cruz, CA). The anti-human p53 (Ab-6) antibody was from EMD Chemicals (Gibbstown, NJ). Human full-length and deleted MDM2 P2 promoter reporters were kind gifts from Dr. J.P. Blaydes [42] (Southampton General Hospital, UK). The MDM2 expression vector was kindly provided by Dr. J. Chen [43] (Moffitt Cancer Center, USA). The pcDNA6/TR and pcDNA4/TO vectors were kindly provided by Dr. X. Chen [44] (University of California, Davis, USA).

### Cell lines and culture

Human breast cancer MCF7, MDA-MB-468, MDA-MB-231, and PC3 cells were obtained from the American Type Culture Collection (Rockville, MD). MDA-MB-231 luciferase-expressing cell line (MDA-MB-231-Luc) was obtained from Caliper Life Sciences (Alameda, CA). All cell culture media contained 10% FBS and 1% penicillin/streptomycin unless otherwise specified. Human MCF7 and MDA-MB-468 cells were grown in MEM media, containing 1 mM non-essential amino acids (NEAA) and Earle's BSS, 1 mM sodium pyruvate and 10 mg/L bovine insulin, and DMEM/F-12 Ham's media (DMEM/F-12 1∶1 mixture), respectively. Human MDA-MB-231 and MDA-MB-231-Luc cells were grown in DMEM medium containing 0.1 mM MEM non-essential amino acids, and 2 mM L-glutamine. PC3 cells were grown in Ham's F-12K medium containing sodium bicarbonate (1.5 mg/mL). Stable PC3 cell lines with MDM2 knockdown were described previously [18]. The inducible MDM2 overexpression (OE) and knockdown (KD) MCF7 cell line was established previously [45] and was grown in DMEM medium containing 7.5 µg/mL blasticidin (Invitrogen, Grand Island, NY) and 200 μg/mL zeocin (Invitrogen, Grand Island, NY).

### Reverse transcription-PCR

Total RNA was extracted using the Trizol reagent (Invitrogen, Grand Island, NY), quantified by UV spectrophotometry, and used to create cDNA with the SuperScript reverse transcription-PCR (RT-PCR) kit from Invitrogen. The PCR coamplification of MDM2 with β-actin was accomplished using the method described previously [17].

### Luciferase assay

Cells were cotransfected with full-length or deleted human MDM2 promoter vectors with the Renilla luciferase reporter as an internal control. The cells were then exposed to 25-OCH_3_-PPD for 24 h. The luciferase activity of the MDM2 promoter reporters was determined using the Dual-Luciferase Reporter Assay System (Promega, Madison, WI), according to the manufacturer's protocol. MDM2 reporter activity was normalized to that for the Renilla luciferase reporter.

### Assays for cell viability, cell proliferation, apoptosis, and cell cycle distribution

The methods used to determine cell viability (MTT assay), proliferation (BrdUrd incorporation assay), apoptosis, and cell cycle distribution were described previously [4], [7]–[9], [19], [20].

### Immunoblotting and immunoprecipitation

Cells were transfected with indicated plasmids in the presence of Lipofectin (Invitrogen, Grand Island, NY) for various times and lysed in NP-40 lysis buffer containing a protease inhibitor mixture from Sigma (St Louis, MO). Cell lysates were used for immunoblotting as described previously [19], [20]. Immunoprecipitation was performed using the indicated antibodies. Beads were washed, and bound proteins were detected by immunoblotting as reported previously [18].

### Wound healing assay

The migratory ability of MDA-MB-231 cells was measured using the wound healing assay. A monolayer of cells was grown to confluence in 6-well plate and at experimental time zero a scratch was made in each well using a pipette tip. The cells were washed twice with FBS-free medium before their subsequent incubation with culture medium in the absence (control) or presence of 25-OCH_3_-PPD at appropriate concentrations. In order to monitor cell movement into the wounded area, five fields of each of the three wounds analyzed per condition were photographed at 0, 8, 24 and 48 h.

### Human breast cancer xenograft models and treatment

The animal study protocol was approved by the Institutional Animal Use and Care Committee of the Texas Tech University Health Sciences Center (IACUC # 10032, PHS Assurance # A 3056-01, USDA Registration # 74-R-0050, REF # 039461). Female athymic pathogen-free nude mice (nu/nu, 4–6 weeks) were purchased from Charles River Laboratories (Wilmington, MA). The MCF7 and MDA-MB-468 human breast cancer xenograft models were established as described previously [19], [20]. All animals were monitored for activity, physical condition, body weight, and tumor growth. The animals bearing human breast cancer xenografts were randomly divided into treatment groups and control group (10–15 mice/group). The control group received the vehicle only. 25-OCH_3_-PPD was dissolved in PEG400: ethanol: saline (57.1: 14.3: 28.6, v/v/v), and was administered by intraperitoneal (i.p.) injection at doses of 5 or 20 mg/kg/d, 5 d/wk for 6 weeks (MCF7) or 4 weeks (MDA-MB-468), respectively. At different times, xenograft tumors were removed and homogenized, and the resultant supernatants were used for Western blotting analysis.

### *In vivo* experimental metastasis assay

MDA-MB-231-Luc cells (1×10^6^) were trypsinized and re-suspended in serum-free medium (100 µL), and intravenously injected into a tail vein of nu/nu mice. These mice were then given luciferase substrate (Caliper, Mountain View, CA) and photographed using IVIS Lumina XR *in vivo* imaging system (Caliper, Mountain View, CA) on Days 1, 8, and 15 for the observation of *in vivo* cancer cell metastasis. 25-OCH_3_-PPD was administered by i.p. injection at 10 mg/kg/d, 5 d/wk for 2 weeks.

### Statistical analysis

The majority of quantitative data in the present study are reported as means ± SD from at least three independent experiments. One-way ANOVA was used to test differences for single group analysis, followed by Tukey's multiple comparisons. Two-way ANOVA was used for grouped analysis of differences followed by Bonferroni post-tests.
